# Sporadic Creutzfeldt-Jakob Disease With Status Epilepticus: Molecular Mechanisms and a Scoping Review of the Literature

**DOI:** 10.7759/cureus.28649

**Published:** 2022-08-31

**Authors:** Bahadar S Srichawla

**Affiliations:** 1 Department of Neurology, UMass Chan Medical School, Worcester, USA

**Keywords:** prion diseases, prion protein, molecular mechanisms, epilepsia partialis continua, non-convulsive, status epilepticus, creutzfeldt jakob disease, infectious disease, neurology, cjd

## Abstract

Creutzfeldt-Jakob disease (CJD) is a rapidly progressing neurodegenerative disorder and is a spongiform encephalopathy. A 59-year-old male presented with subacute-onset worsening encephalopathy and was found to be in non-convulsive status epilepticus (NCSE) requiring intubation and a midazolam infusion for refractory seizures. Electroencephalogram (EEG) revealed triphasic repeats with focal epileptogenic originating from the left parietal region. The patient continued to have up to 25-40 non-convulsive seizures per day. Cerebrospinal fluid (CSF) analysis revealed elevated 14-3-3 and tau protein. A real-time quaking-induced conversion assay in CSF was positive. The patient was diagnosed with probable sporadic CJD based on criteria from the Centers for Disease Control. Supportive treatment was provided.

Cellular prion protein (PrP^C^) plays an important role in myelination of the peripheral nervous system, regulation of the neuronal membrane, and circadian rhythm. The molecular mechanisms of CJD involve the catalyzation of the physiological PrP^C ^into the pathological prion protein (PrP^Sc^). This post-translational change in conformation leads to the generation of PrP^Sc ^and is involved in spongiform encephalopathies. Mechanisms of neurodegeneration include astrocytosis, neuronal apoptosis, and amyloid plaque formation. A scoping literature review was conducted in three databases on cases of CJD with SE. A total of 13 cases are identified that include the type of CJD and the morphology of the seizures. NCSE is the most prevalent form of SE in patients with CJD.

## Introduction

Creutzfeldt-Jakob disease (CJD) is a rare neurodegenerative disease mediated by a pathological prion protein (PrP^Sc^). CJD is characterized in four distinct variants: sporadic Creutzfeldt-Jakob disease (sCJD), familial Creutzfeldt-Jakob disease (fCJD), variant Creutzfeldt-Jakob disease (vCJD), and iatrogenic Creutzfeldt-Jakob disease (iCJD). Most cases of CJD belong to the sporadic variant (85-95%). CJD is more commonly seen in the elderly population with a mean age in the seventh decade of life. CJD affects about one person per million worldwide and carries a devastating prognosis [1]. Seizures rarely present as an early symptom of CJD. Status epilepticus (SE) is characterized by convulsive status epilepticus (CSE), non-convulsive status epilepticus (NCSE), and epilepsia partialis continua (EPC).

Here, a 59-year-old male is presented who developed NCSE (25-40 non-convulsive seizures per day), secondary to the neurodegenerative consequences of CJD evident based on the presence of 14-3-3, tau protein, and real-time quaking-induced conversion (RT-QuIC). An overview of various molecular mechanisms of neurodegeneration in CJD is included. A scoping review of the literature was conducted to analyze the most prevalent form of SE in patients with CJD. 

## Case presentation

A 59-year-old male with no significant medical history came to the emergency department with a rapidly progressing subacute-onset encephalopathy, and abnormal jerking movements of the upper extremity. The patient was brought in at the request of a family member for evaluation. The patient had a sedentary desk job and had no recent sick contacts or infections. Vital signs on presentation were significant for a blood pressure of 145/90 mmHg, heart rate of 79 beats per minute, body temperature of 35.6 Celsius, and 98% oxygen saturation on room air measured via pulse oximetry. The patient was alert and oriented to person, place, and time on neurologic examination. A score of 13 was noted on the Montreal Cognitive Assessment (MoCA) significant for moderate cognitive impairment. A wide-based ataxic gait was observed. Intermittent myoclonic jerks were observed in the upper and lower extremities. A comprehensive blood count, comprehensive metabolic panel, hepatitis panel, and human immunodeficiency virus (HIV) were negative. Further diagnostic testing for heavy metal poisoning including lead, mercury, manganese, and copper was negative.

**Figure 1:**
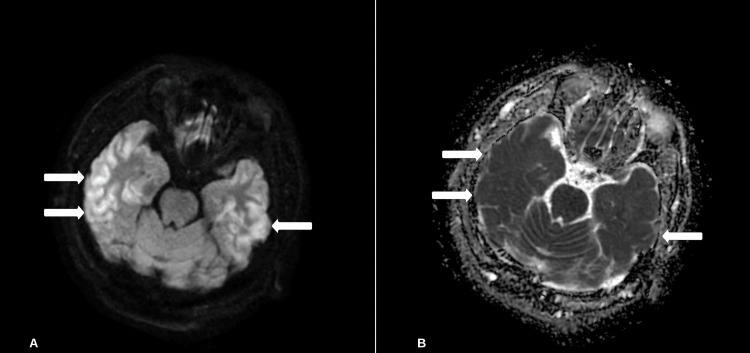
MRI of the brain axial view of DWI and ADC (A) Axial DWI sequences reveal significant bilateral temporal lobe diffusion restriction in a cortical pattern. (B) Correlating ADC sequence. DWI: diffusion-weighted imaging; ADC: apparent diffusion coefficient.

A computerized tomography (CT) scan of the head revealed no abnormalities, and a CT angiogram of the head and neck was unremarkable. Magnetic resonance imaging (MRI) of the brain revealed T2 hyperintensities and restricted diffusion on diffusion-weighted imaging (DWI) of the bilateral temporal gyri, in a cortical pattern consistent with cortical ribboning. These findings are concerning for an interictal state or neurodegenerative process (Figure 1).

**Figure 2:**
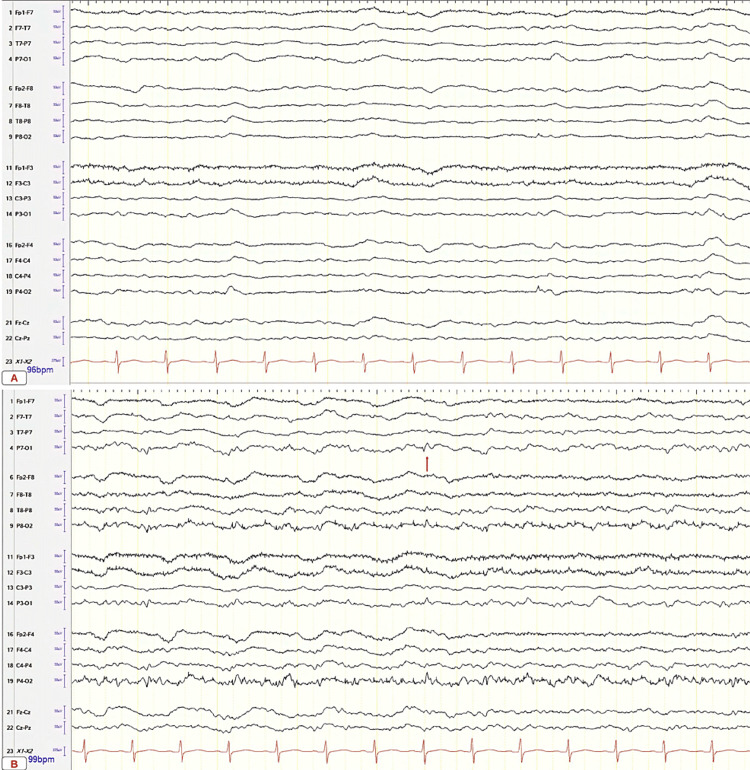
Continuous 21-electrode EEG (international 10-20 system). Single-channel EKG recording. (A) Moderate to severe diffuse generalized slowing and disorganization consistent with cerebral dysfunction and encephalopathy. (B) Focal non-convulsive electrographic seizures from the left parietal region. Electrographic seizures lasting >30 seconds. Approximately 25-40 seizure episodes were observed on average in a 24-hour period. EEG findings significant for occasional generalized periodic discharges more predominant in the bifrontal leads with triphasic morphology typically at 1-2.0 Hz. Occasional spikes and sharp waves occur approximately once per minute. Intermittent non-time locked myoclonic jerks of the right upper extremity. Predominant delta and theta wave background. EEG: electroencephalogram.

The patient was continuously placed on an electroencephalogram, revealing generalized slowing, triphasic repeats with focal epileptogenic activity from the left parietal region (Figure 2). The patient was started on antiepileptic drugs (AEDs), including lorazepam and fosphenytoin. Despite aggressive medical treatment, the patient remained in NCSE with up to 40 episodes per day. The patient was subsequently intubated for airway protection and started on a midazolam infusion. On day 7 of hospitalization, the patient was free from seizures and extubated, and started on an oral AED regimen of levetiracetam 2000 mg twice a day, clobazam 5 mg twice a day, and lacosamide 300 mg per day.

Further diagnostic testing with a comprehensive cerebrospinal fluid (CSF) examination that included cell count, immunoglobulin G (IgG), IgG index, autoimmune, meningitis, encephalitis, and the paraneoplastic panel was negative. Significant findings included an elevated CSF protein at 55 mg/dL (18-45), the 14-3-3 protein was 38592 AU/mL (0-1149), the tau protein was 18976 pg/mL (<30-1999), and the RT-QuIC assay was positive. A diagnosis of probable sCJD was made based on the CDC criteria. Repeat MRI approximately five weeks after initial presentation revealed persistent but improved diffusion restriction in DWI and hyperintensities in T2-weighted imaging with improvement in gyral swelling.

**Figure 3:**
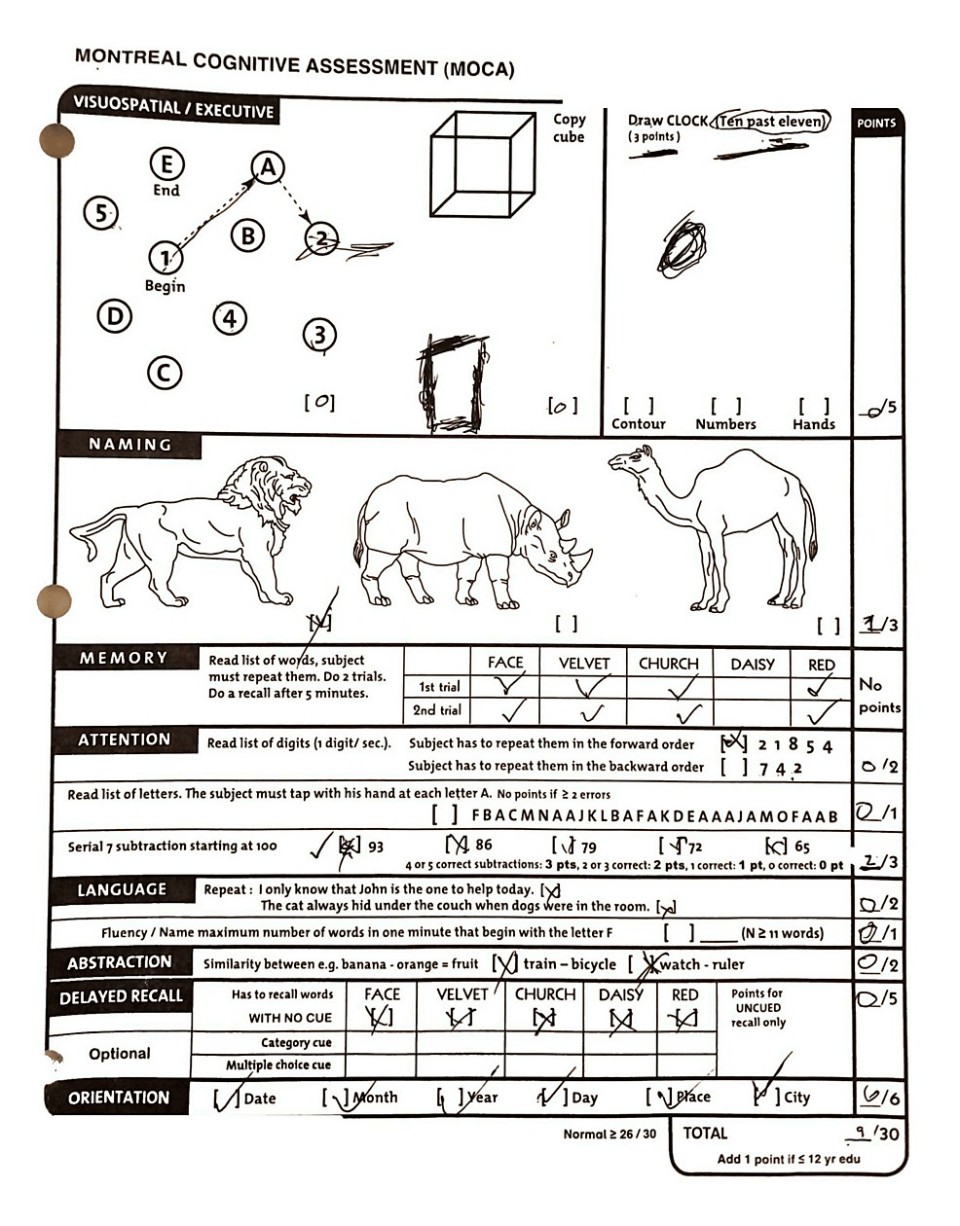
Montreal cognitive assessment (MoCA) completed at discharge. MoCA© (Version November 7, 2004) score of 9/30 reveals severe cognitive impairment. Disclosure: Written permission obtained from MoCA Test Inc

Throughout admission, the patient continued to have a worsening mental state consistent with the rapidly progressing course of CJD. The patient was offered supportive care and was discharged to a long-term care facility. On the day of discharge, a repeat MoCA© was completed with a score of 9 (Figure 3). The patient was discharged on an AED regimen including levetiracetam, clobazam, and lacosamide.

## Discussion

**Figure 4:**
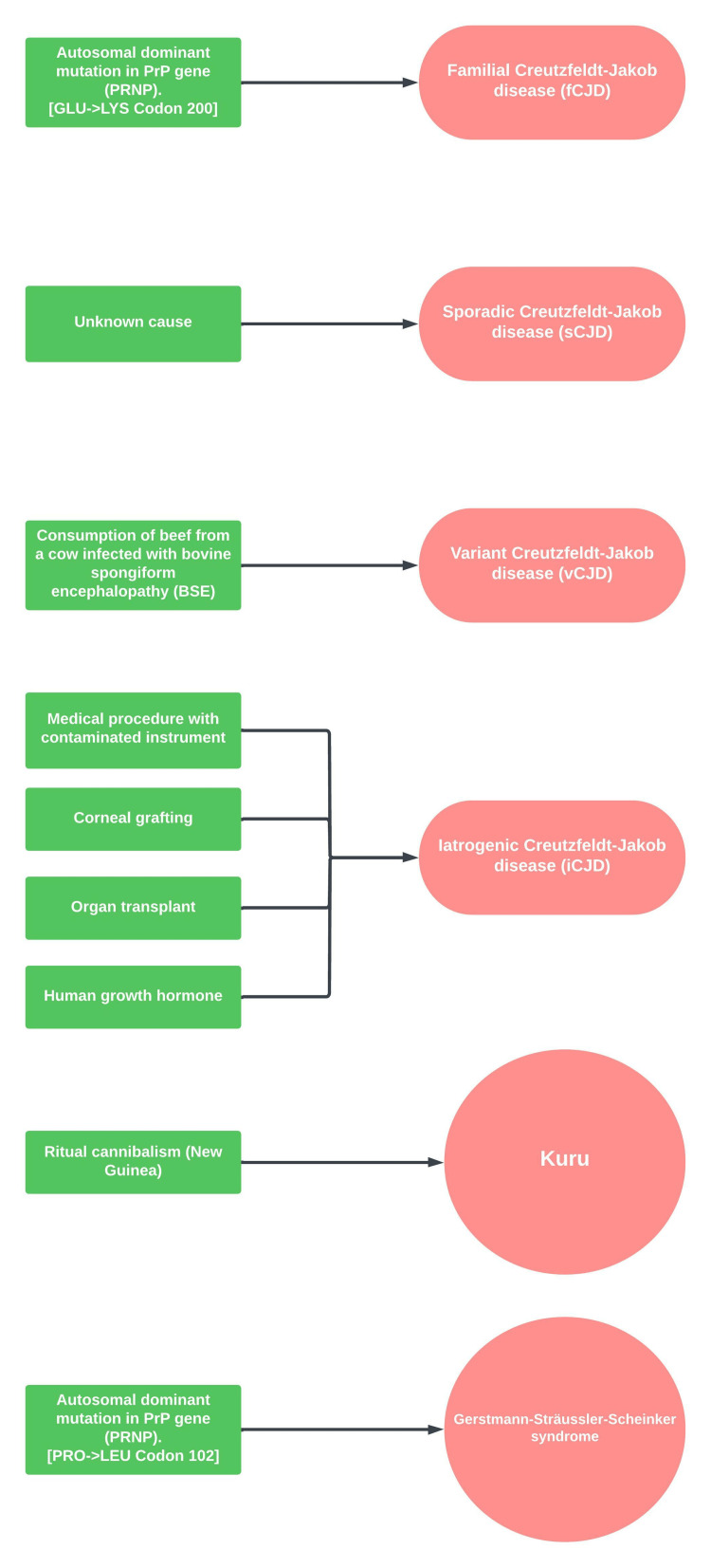
Overview of various prion diseases and their causes. sCJD: sporadic Creutzfeldt-Jakob disease; fCJD: familial Creutzfeldt-Jakob disease; iCJD: iatrogenic Creutzfeldt-Jakob disease; vCJD: variant Creutzfeldt-Jakob disease; PRP: prion protein; PRNP: prion protein gene; PRO: proline; LEU: leucine; LYS: lysine; GLU: glutamic acid. Image Credits: Bahadar S. Srichawla

CJD belongs to the family of spongiform encephalopathies and has a devastating prognosis. Four different variants for CJD with varying etiologies are described. The familial variant occurs due to a missense mutation in the prion protein (PrP) gene (PRNP) at codon 200. The iatrogenic variant can occur secondary to neurosurgical procedures with contaminated equipment, corneal grafting, and organ transplant among other causes. Variant CJD can occur secondary to the consumption of beef infected with bovine spongiform encephalopathy. The sporadic variant is the most common form of CJD and accounts for approximately 85% of all reported cases. Although the precise cause is unknown, it is hypothesized that it occurs due to the catalyzation of the cellular prion protein (PrP^C^) into the pathological variant (PrP^Sc^) [2]. Other lesser-known causes of spongiform encephalopathies are Kuru and Gerstmann-Sträussler-Scheinker syndrome. Kuru occurs due to cannibalism and is seen in the tribes of Papa New Guinea. Gerstmann-Sträussler-Scheinker syndrome occurs due to an autosomal dominant gene mutation of the PRNP gene at codon 102 (Figure 4) [3].

Analysis of the CSF plays a cardinal role in the diagnosis of CJD. Elevated 14-3-3 and tau proteins are supportive in the diagnosis of CJD and are markers of neurodegeneration. Elevation of these proteins along with the clinical syndrome of rapidly progressing encephalopathy, cerebellar signs, myoclonus or akinetic mutism, as well as sharp wave complexes on EEG and cortical involvement on MRI meets the criteria for probable CJD as seen in our case. The RT-QuIC assay provides greater sensitivity and specificity in the diagnosis of CJD. A definitive diagnosis of CJD can only be made by histopathological analysis by autopsy. Common histopathological findings include vacuoles within the gray matter that are significant for spongiform changes [4].

Neuroimaging plays a lesser role in the diagnosis of CJD. Cortical lesions are more commonly seen and may appear as a "cortical ribbon" sign on MRI. The involvement of the thalamus and basal ganglia can be found in CJD; however, limbic encephalitis should be considered [5]. Lesions involving the corpus callosum are more consistent with an autoimmune phenomenon such as Susac syndrome [6]. Many cases of CJD often present with diffusion restriction within the cerebral cortex and basal ganglia. Neuroinfectious causes of subcortical lesions include flaviviruses such as the West Nile virus [7].

**Figure 5:**
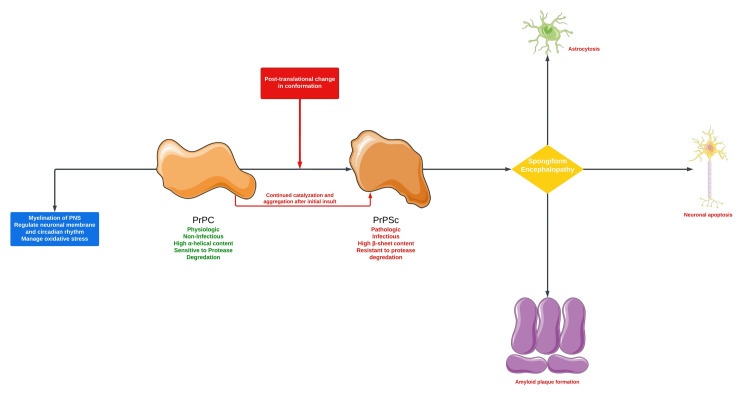
Molecular mechanisms associated with spongiform encephalopathy PrP^C^: cellular prion protein; PrP^Sc^: pathological prion protein. Parts of the figure were drawn using pictures from Servier Medical Art. Servier Medical Art by Servier is licensed under a Creative Commons Attribution 3.0 Unported License (https://creativecommons.org/licenses/by/3.0/). Image credits: Bahadar S. Srichawla.

Although the exact mechanism of sCJD is not known, it is the pathogenesis that is believed to involve a post-translational change in the confirmation of PrP^C^ into PrP^Sc^. The cellular prion protein plays an important role in neuronal cell membrane regulation, circadian rhythm, and myelination of axons in the peripheral nervous system. This initial insult leads to a catalyzing cascade propagating further insult. The PrP^Sc^ has a high beta-sheet content in comparison to the predominantly alpha-helical-shaped PrP^C^. The PrP^Sc^ causes neuronal apoptosis, astrocytosis, and the accumulation of amyloid plaque as a direct result. Figure 5 provides an overview of the molecular pathogenic mechanisms associated with CJD [8].

Literature review

**Figure 6:**
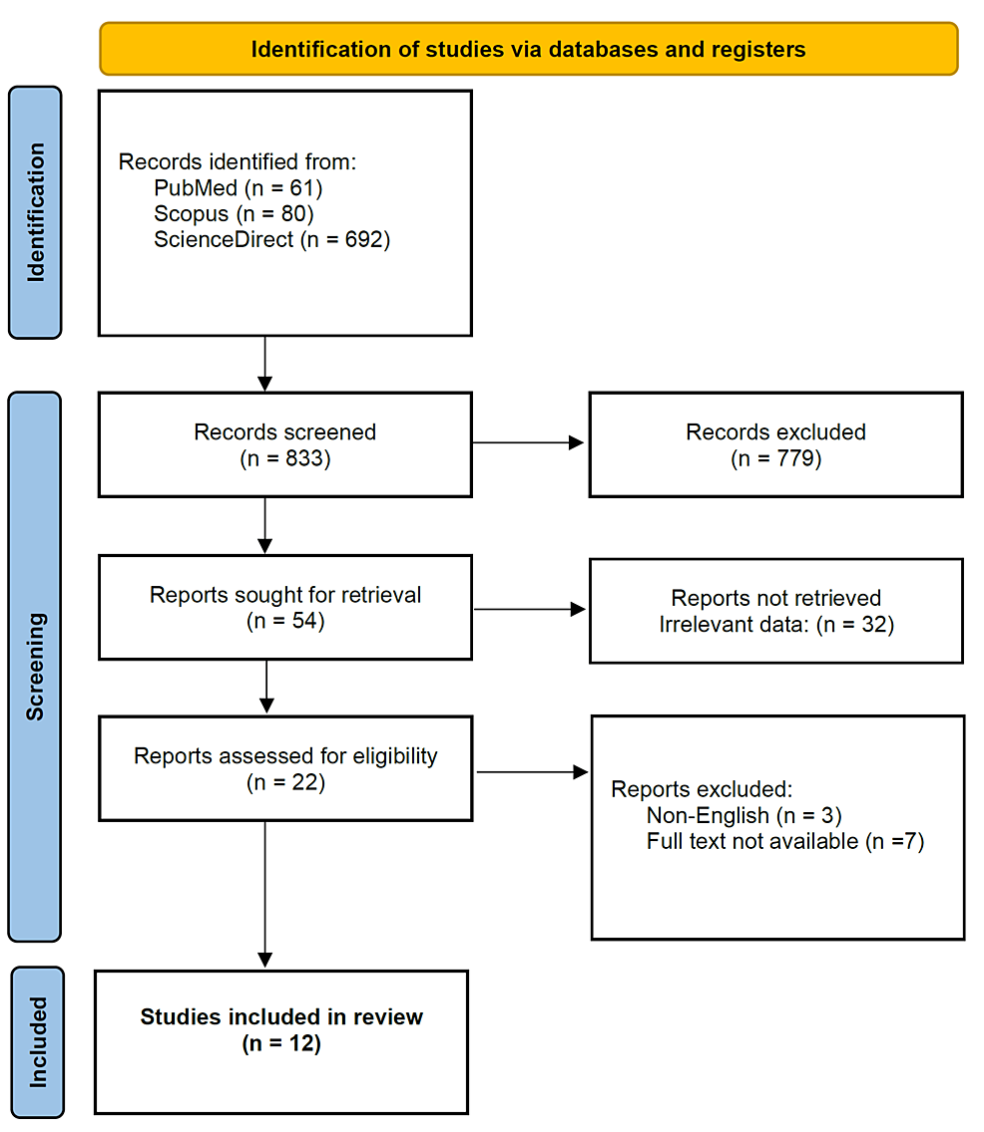
Flow diagram depicting study selection for the scoping literature review.

This scoping literature review aimed to identify cases of CJD with SE (convulsive, non-convulsive, and EPC). A comprehensive literature search was conducted in PubMed/PubMedCentral/MEDLINE, Scopus, and ScienceDirect databases. The following search term was utilized: '(“Creutzfeldt-Jakob disease” OR “Creutzfeldt-Jakob”) AND (“status epilepticus” OR "epilepsia partialis continua")'. All searches were completed on July 14th, 2022. Only case reports/series were included. Only cases of probable and confirmed CJD were included. Exclusion criteria included non-English articles, and articles with the full text unavailable. Records lacking date on seizure semiology were also removed. A total of 833 search results were obtained. Seven hundred and seventy-nine results were excluded because they were not peer-reviewed journal case reports/series relevant to the question. A total of 54 articles were examined for retrieval and 32 articles were excluded due to irrelevant data. A total of 22 records were assessed for eligibility and 10 were removed either for being non-English articles or not having the full text available for analysis. A total of 12 records were included in the review, with 13 cases identified. A flow diagram is provided to visualize study selection based on the criteria above (Figure 6). Results are summarized in Table 1. Data analysis and visualization were completed on GraphPad Prism 9.0.0 for Windows, GraphPad Software, San Diego, California, USA. The scoping review was registered on Open Science Framework (Registration ID: 10.17605/OSF.IO/FD38J). 

**Table 1:** Cases of SE identified in confirmed or probable cases of CJD. PLED: periodic lateralized epileptiform discharges; SE: status epilepticus; CSE: convulsive status epilepticus; NCSE: non-convulsive status epilepticus; CJD: Creutzfeldt-Jakob disease; EPC: epilepsia partialis continua; CSF: cerebrospinal fluid.

	Author	Age	Gender	CJD Variant	Seizure Morphology
1	Aiguabella et al. (2010) [9]	44	Male	Confirmed sporadic CJD. Autopsy confirmed.	NCSE: Continuous generalized periodic epileptiform discharges with a frequency of 1.5 Hz.
2	Coric et al. (2001) [10]	57	Male	Probable sporadic CJD. Elevated biomarkers in CSF.	NCSE: Diffuse spike-wave complex discharges at a rate of 2-3 Hz.
3	Espinosa et al. (2010) [11]	64	Female	Confirmed sporadic CJD. Autopsy confirmed.	NCSE: 1-1.5 Hz periodic sharp wave discharges maximal on the right temporal region.
4	Fernandez-Torre et al. (2004) [12]	75	Female	Confirmed sporadic CJD. Autopsy confirmed.	NCSE: Continuous diffuse spikes, rhythmic sharp waves, and sharp-and-slow wave complexes. Repeat EEG showed pseudo-periodic negative or positive-negative slow waves localized in the right frontal region.
5	Hsiao et al. (2019) [13]	83	Male	Probable sporadic CJD. Elevated 14-3-3 in CSF.	EPC: Rhythmic epileptiform discharges arising from the right hemisphere and then spreading to the contralateral hemisphere. An interictal EEG revealed right-sided lateralized periodic discharges with maximum amplitude in the right mid-frontal region.
6	Lowden et al. (2008) [14]	49	Female	Familial CJD. Biopsy proven.	EPC: PLEDs with continuous spike-wave discharges seen predominantly from the right centroparietal region.
7	Mahboob et al. (2018) [15]	60	Male	Probable sporadic CJD. Elevated 14-3-3 and tau protein. RT-QuIC positive.	NCSE: Persistent epileptogenic activity with bilateral hemispheric discharges.
8	Miyake et al. (2018) [16]	82	Female	Confirmed sporadic CJD. Autopsy confirmed.	NCSE: Spikes in the left parietal region, and slow wave bursts in the bilateral frontal areas. Evolving into generalized periodic discharges.
9	Neufeld et al. (2003) [17]	62	Male	Confirmed sporadic CJD. Autopsy confirmed.	CSE: Diffuse slowing, suppression of background activity, and bilateral synchronous periodic epileptiform discharges.
10	Rakitin et al. (2018) [18]	74	Female	Confirmed sporadic CJD. Autopsy confirmed.	NCSE: Pseudoperiodic lateralized epileptiform discharges over the right hemisphere with a frequency of 2-3 Hz.
11	Rees et al. (1999) [19]	58	Female	Confirmed sporadic CJD. Autopsy confirmed.	NCSE: Continuous variable amplitude sharp waves in all areas, although with a right-sided emphasis, with a repetitive appearance up to 2 per second.
12	Rees et al. (1999) [19]	68	Male	Probable sporadic CJD. CSF biomarkers present with clinical symptoms.	NCSE: PLEDs, more marked on the left side. Repeat EEG 3 weeks later again showed frequent predominantly left-sided epileptiform discharges.
13	Rossetti et al. (2007) [20]	74	Female	Probable sporadic CJD. Elevated 14-3-3 protein in CSF.	NCSE: Isorganized alpha–theta background with intermittently superimposed irregular spikes (about 2-3 Hz), non-reactive, slightly predominating on the right.

**Figure 7:**
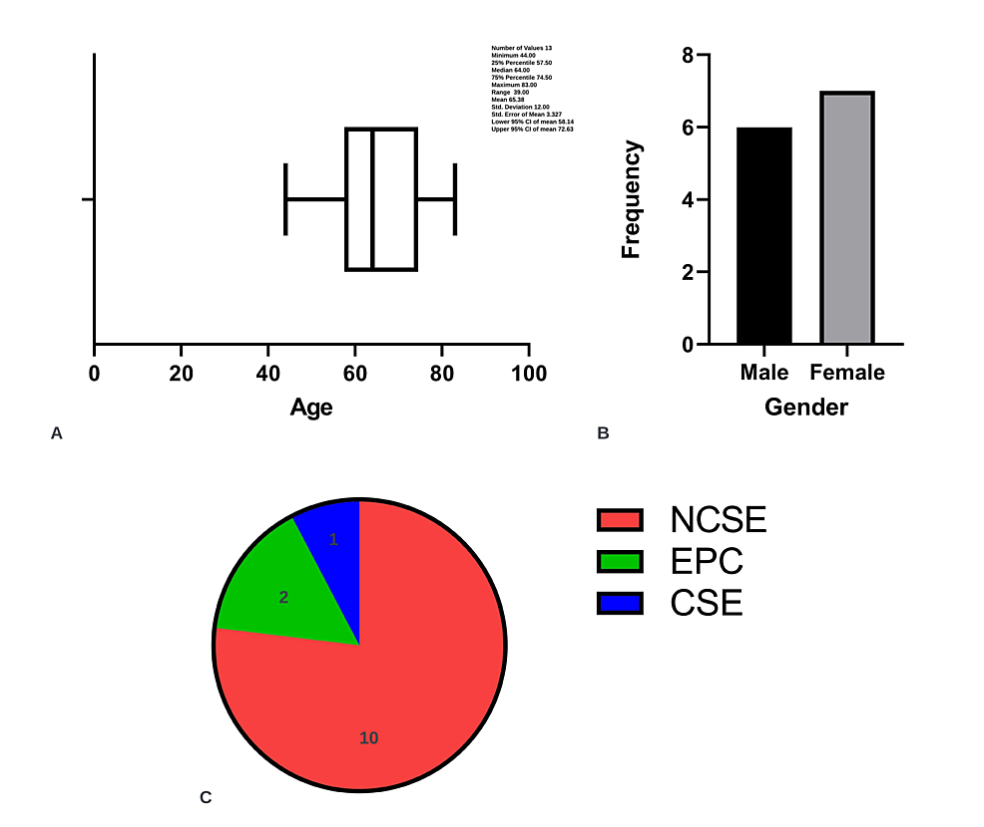
Age, gender, and type of status epilepticus identified (A) Mean age of diagnosis at 65.3 years (SD 12.0, SEM: 3.3). (B) Seven female and six male patients were identified. (C) Distribution of cases including NCSE, CSE, and EPC. SEM: standard error of the mean; SD: standard deviation; CJD: Creutzfeldt-Jakob disease; SE: status epilepticus; NCSE: non-convulsive status epilepticus; CSE: convulsive-status epilepticus; EPC: epilepsia partialis continua.

**Figure 8:**
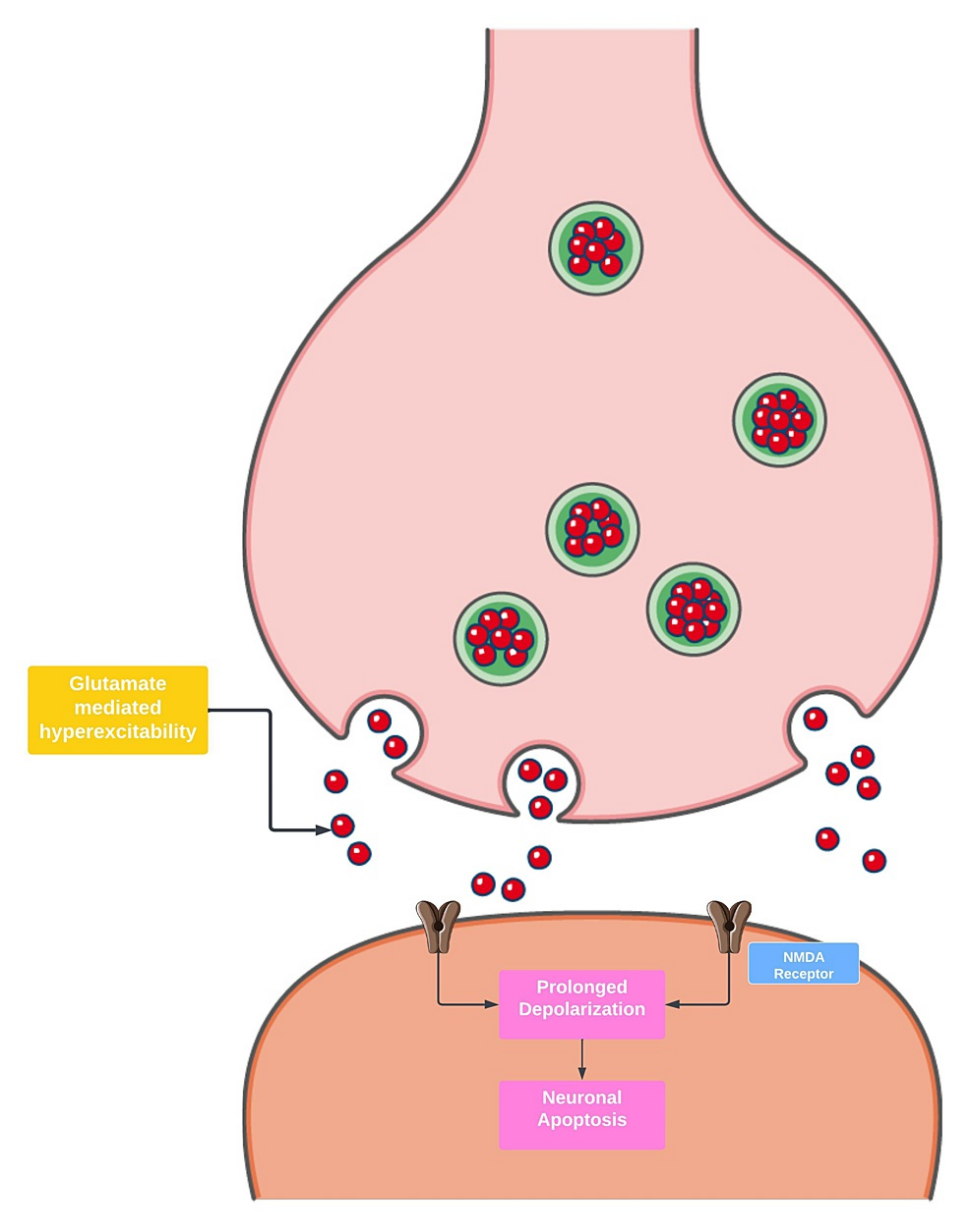
Mechanisms of synaptic glutamate excitotoxicity NMDA: N-methyl-D-aspartate. Parts of the figure were drawn using pictures from Servier Medical Art. Servier Medical Art by Servier is licensed under a Creative Commons Attribution 3.0 Unported License (https://creativecommons.org/licenses/by/3.0/). Image credits: Bahadar S. Srichawla.

A total of 13 cases are included in the scoping review based on the inclusion and exclusion criteria. A total of 12 cases of sporadic CJD (probable or confirmed) are identified and one case of familial CJD [14]. The most prevalent form of SE was the non-convulsive type accounting for approximately 75% of reported cases. The mean age of reported cases is 65.38 (standard deviation [SD]: 12.00, standard error of the mean [SEM]: 3.3). The data are normally distributed and passed the Shapiro-Wilk test of normality. Seven cases (53.8%) are reported in females, and six cases (46.1%) are reported in males (Figure 7). Two cases of EPC and one case of CSE are reported. Epileptic seizures are observed in approximately 15% of patients with sCJD and are typically a late presenting feature. SE is defined as the presence of seizures for more than 5 minutes, or more than one seizure in a 5-minute period before the baseline level of consciousness is obtained. Mechanisms of SE are believed to involve continuous excitatory input at postsynaptic neurons, and glutamatergic excitability is often implicated (Figure 8).

In the described case, myoclonic jerks were not time-locked with epileptiform discharges observed in the left hemisphere. However, the administration of benzodiazepines had a positive electrophysiologic effect on the morphology of seizures consistent with NCSE. Many of the radiographic findings of CJD coincide with NCSE. Both processes can show hyperintensities within the cerebral cortex. The additional findings of basal ganglia involvement in neuroimaging are more consistent with CJD. However, not all cases of CJD have involvement of the basal ganglia, making an accurate diagnosis of either condition difficult based solely on neuroimaging. 14-3-3 and the tau proteins are sensitive biomarkers of neurodegeneration and can be elevated due to SE. A positive result on the RT-QuIC assay provides the most specific test for the diagnosis of CJD, as it directly measures the presence of the PrP^Sc^. Histopathological analysis of brain tissue often during postmortem autopsy is the only way to confirm the diagnosis of CJD [4].

## Conclusions

CJD is a rare neurodegenerative disease that is classified by four variants. The case of probable sporadic CJD is described with a clinical course complicated with NCSE. A scoping review of the literature was conducted revealing most cases of SE in CJD are dominated by NCSE. Fewer cases of EPC and CSE are seen in the literature, respectively. Molecular mechanisms associated with CJD involve the continuous catalyzation of the PrP^C^ into the pathological variant. PrP^Sc^ exerts spongiform changes within the CNS through astrocytosis, neuronal degradation, and amyloid plaque accumulation among other mechanisms.
